# Porcine IKKε is involved in the STING-induced type I IFN antiviral response of the cytosolic DNA signaling pathway

**DOI:** 10.1016/j.jbc.2023.105213

**Published:** 2023-09-01

**Authors:** Jia Luo, Qi Cao, Jiajia Zhang, Sen Jiang, Nengwen Xia, Shaohua Sun, Wanglong Zheng, Nanhua Chen, Francois Meurens, Jianzhong Zhu

**Affiliations:** 1 Comparative Medicine Research Institute, Yangzhou University, Yangzhou, China; 2 College of Veterinary Medicine, Yangzhou University, Yangzhou, China; 3 Joint International Research Laboratory of Agriculture and Agri-Product Safety, Yangzhou University, Yangzhou, China; 4 Jiangsu Co-Innovation Center for Prevention and Control of Important Animal Infectious Diseases and Zoonoses, Yangzhou University, Yangzhou, China; 5 Faculty of Veterinary Medicine, Swine and Poultry Infectious Diseases Research Center, University of Montreal, St Hyacinthe, Quebec, Canada; 6 Department of Veterinary Microbiology and Immunology, Western College of Veterinary Medicine, University of Saskatchewan, Saskatoon, Saskatchewan, Canada

**Keywords:** porcine, cGAS–STING, IKKε, TBK1, IFN, antiviral

## Abstract

The cyclic GMP–AMP synthase and stimulator of interferon (IFN) genes (cGAS–STING) pathway serves as a crucial component of innate immune defense and exerts immense antiviral activity by inducing the expression of type I IFNs. Currently, STING-activated production of type I IFNs has been thought to be mediated only by TANK-binding kinase 1 (TBK1). Here, we identified that porcine IKKε (pIKKε) is also directly involved in STING-induced type I IFN expression and antiviral response by using IKKε^−/−^ porcine macrophages. Similar to pTBK1, pIKKε interacts directly with pSTING on the C-terminal tail. Furthermore, the TBK1-binding motif of pSTING C-terminal tail is essential for its interaction with pIKKε, and within the TBK1-binding motif, the leucine (L) 373 is also critical for the interaction. On the other hand, both kinase domain and scaffold dimerization domain of pIKKε participate in the interactions with pSTING. Consistently, the reconstitution of pIKKε and its mutants in IKKε^−/−^ porcine macrophages corroborated that IKKε and its kinase domain and scaffold dimerization domain are all involved in the STING signaling and antiviral function. Thus, our findings deepen the understanding of porcine cGAS–STING pathway, which lays a foundation for effective antiviral therapeutics against porcine viral diseases.

The innate immune response serves as the first-line defense against infections and utilizes multiple pattern recognition receptors (PRRs) to monitor the invading pathogens by sensing the pathogen-associated molecular patterns and damage-associated molecular patterns (1, 2). The innate immune PRRs include Toll-like receptors, cytosolic RIG-I-like receptors, NOD-like receptors, C-type lectin-like receptors, and cytosolic DNA receptors (CDRs). Once activated, PRRs trigger cell signaling cascades to initiate downstream transcriptional induction of interferons (IFNs), proinflammatory cytokines, and transcription-independent protease activation, orchestrating innate immune responses (2, 3).

Among the various PRRs, nucleic acid sensing RIG-I-like receptors and CDRs play a critical role in the cellular antiviral responses owing to the potent induction of IFN response during virus infection (4, 5). As a CDR member and a nucleotide transferase, the cGAS utilizes GTP and ATP as substrates to synthesize the second messenger 2′3′-cGAMP upon activation by the agonist DNA (6). The 2′3′-cGAMP in turn binds and activates the signaling adaptor STING on the endoplasmic reticulum (ER), which is subjected to oligomerization and migration from ER to Golgi *via* the ER–Golgi intermediate compartment (7, 8). Upon activation, STING recruits TBK1 through the [(D/E)xPxPLR(S/T)D] motif in its C-terminal tail (CTT) (8, 9). The recruited TBK1 is self-transactivated and phosphorylates conserved serine (S) located in the pLxIS motif of STING CTT (10), which allows the recruiting of the transcription factor interferon regulatory factor 3 (IRF3) and its phosphorylation by nearby TBK1 (11). The phosphorylated IRF3 forms a dimer and translocates into cell nucleus to drive the transcription of type I IFNs and the numerous IFN-stimulated genes (ISGs), inducing a robust antiviral state (12, 13). STING also activates NF-κB to a low extent *via* the IKKα/β/γ complex to synergize with IRF3 inducing the expression of IFNs and to produce proinflammatory cytokines (7).

It has been widely accepted that TBK1 is predominantly required for cGAS–STING pathway–induced IRF3 and NF-κB activation and downstream cytokine production (10, 11, 14). However, several lines of evidence demonstrated that STING may induce IFN production *via* both TBK1 and IKKε participation. First, the TBK1–IKKε kinase complex plays an essential role in the direct phosphorylation of the transcription factors IRF3 and IRF7, allowing the activation of IFN gene transcription (15, 16). Second, TBK1 and IKKε have a similar domain composition and share the same substrate consensus phosphorylation motif, and the upstream activation mechanisms and the downstream targets for both kinases are highly similar or identical (17, 18, 19). Third, it has been recently shown that IKKε participates redundantly with TBK1 in the STING-induced IFN response as well as NF-κB response (20, 21). Finally, our recent work reported that African swine fever virus pS273R disturbs the STING-mediated antiviral function by targeting and impairing the function of IKKε to achieve the immune evasion (22). These results raise the vital question whether and how IKKε is involved in the STING-mediated type I IFN response and antiviral activity.

Here, we identified that porcine IKKε (pIKKε) is involved in porcine cGAS–STING pathway–induced type I IFN response and antiviral activity. It seemed that pIKKε and pTBK1 are cooperative in pSTING-induced type I IFN expression, whereas both are redundant in pSTING-mediated NF-κB response. We further demonstrated that pIKKε interacts with pSTING and IRF3 in a way highly similar to pTBK1. Our findings are essential for understanding the molecular mechanism of porcine cGAS–STING pathway and laying a foundation to formulate future therapeutic strategies against porcine viral diseases.

## Results

### pIKKε positively regulates pSTING-induced type I IFN response

**Figure 1:**
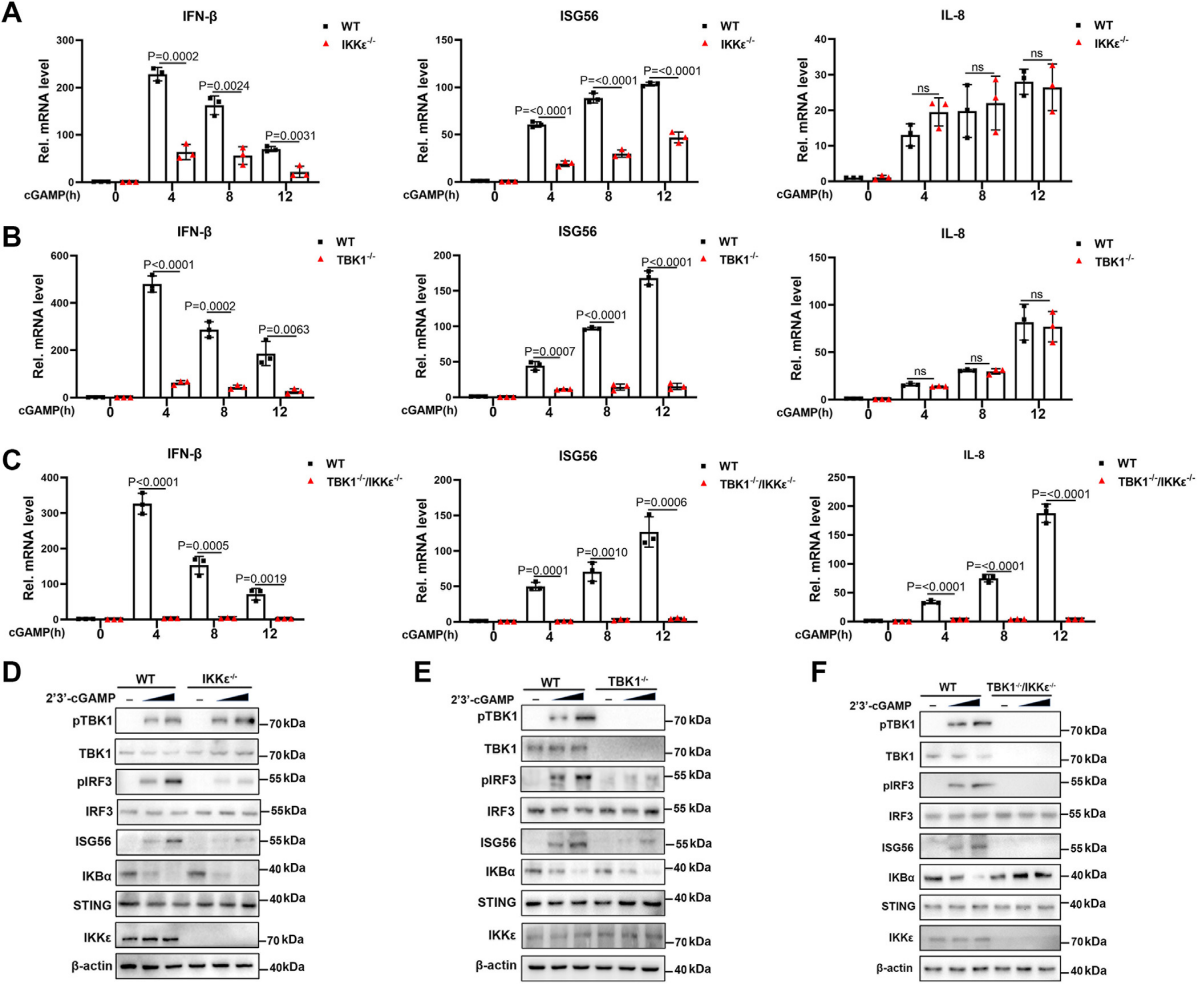
**pIKKε positively regulates pSTING-induced type I IFN response.***A*–*C*, the pIKKε^−/−^ 3D4/21 cells, pTBK1^−/−^3D4/21 cells, pTBK1^−/−^/pIKKε^−/−^3D4/21 cells, and control 3D4/21 cells in 24-well plates (3 × 10^5^ cells/well) were transfected with 2′3′-cGAMP (2 μg/ml) by using Lipofectamine 2000 for 0 to 12 h, respectively. The harvested cells were measured by RT–qPCR for downstream gene expressions. *D*–*F*, pIKKε^−/−^ 3D4/21 cells (*D*), pTBK1^−/−^ 3D4/21 cells (*E*), pIKKε^−/−^pTBK1^−/−^ 3D4/21 cells (*F*), and 3D4/21 cells in 24-well plate (3 × 10^5^ cells/well) were transfected with 2′3′-cGAMP (1 and 2 μg/ml) for 8 h. The harvested cells were detected by Western blotting with the indicated antibodies. IFN, interferon; pIKKε, porcine IKKε; qPCR, quantitative PCR; STING, stimulator of IFN genes.

TBK1 and IKKε are two important upstream kinases essential for the IRF3 phosphorylation and antiviral immunity (17, 21); however, whether IKKε is required for pSTING-mediated type I IFN production is still not clear. To verify this, we utilized pIKKε^−/−^ 3D4/21 macrophages together with normal control 3D4/21 cells stimulated with STING-specific agonist 2′3′-cGAMP. The results showed that the 2′3′-cGAMP-stimulated gene transcription of IFNβ and ISG56 was significantly reduced in IKKε^−/−^ cells, regardless of the normal interleukin-8 gene transcription in IKKε^−/−^ cells, compared with those in normal 3D4/21 cells (Fig 1*A*). The pTBK^−/−^ and pTBK^−/−^IKKε^−/−^ 3D4/21 cells were also used for stimulation with 2′3′-cGAMP and measured for downstream gene inductions. The results showed that IFNβ and ISG56 gene expressions were largely decreased, whereas interleukin-8 expression was normal in pTBK1^−/−^ macrophages (Fig. 1*B*). On the other hand, all three gene expressions almost disappeared in pTBK^−/−^IKKε^−/−^ macrophages (Fig. 1*C*).

We next treated pIKKε^−/−^ cells, pTBK1^−/−^ cells, pTBK^−/−^IKKε^−/−^, and WT 3D4/21 cells with 2′3′-cGAMP to detect the expression of downstream signaling proteins. Of note, upon stimulation, pIKKε^−/−^ cells exhibited reduced IRF3 phosphorylation and ISG56 expression compared with WT cells, whereas IκBα degradation remained normal (Fig. 1*D*). Moreover, we also observed reduced IRF3 phosphorylation and ISG56 expression but normal IκBα degradation in stimulated pTBK1^−/−^ cells (Fig. 1*E*). In addition, the IRF3 phosphorylation, ISG56 expression, and IκBα degradation totally disappeared in stimulated pTBK1^−/−^pIKKε^−/−^ cells, relative to WT cells (Fig. 1*F*). These data suggested that, similar to pTBK1, pIKKε also participates in pSTING-mediated type I IFN response.

### pIKKε deficiency impairs pSTING-mediated antiviral activity

**Figure 2:**
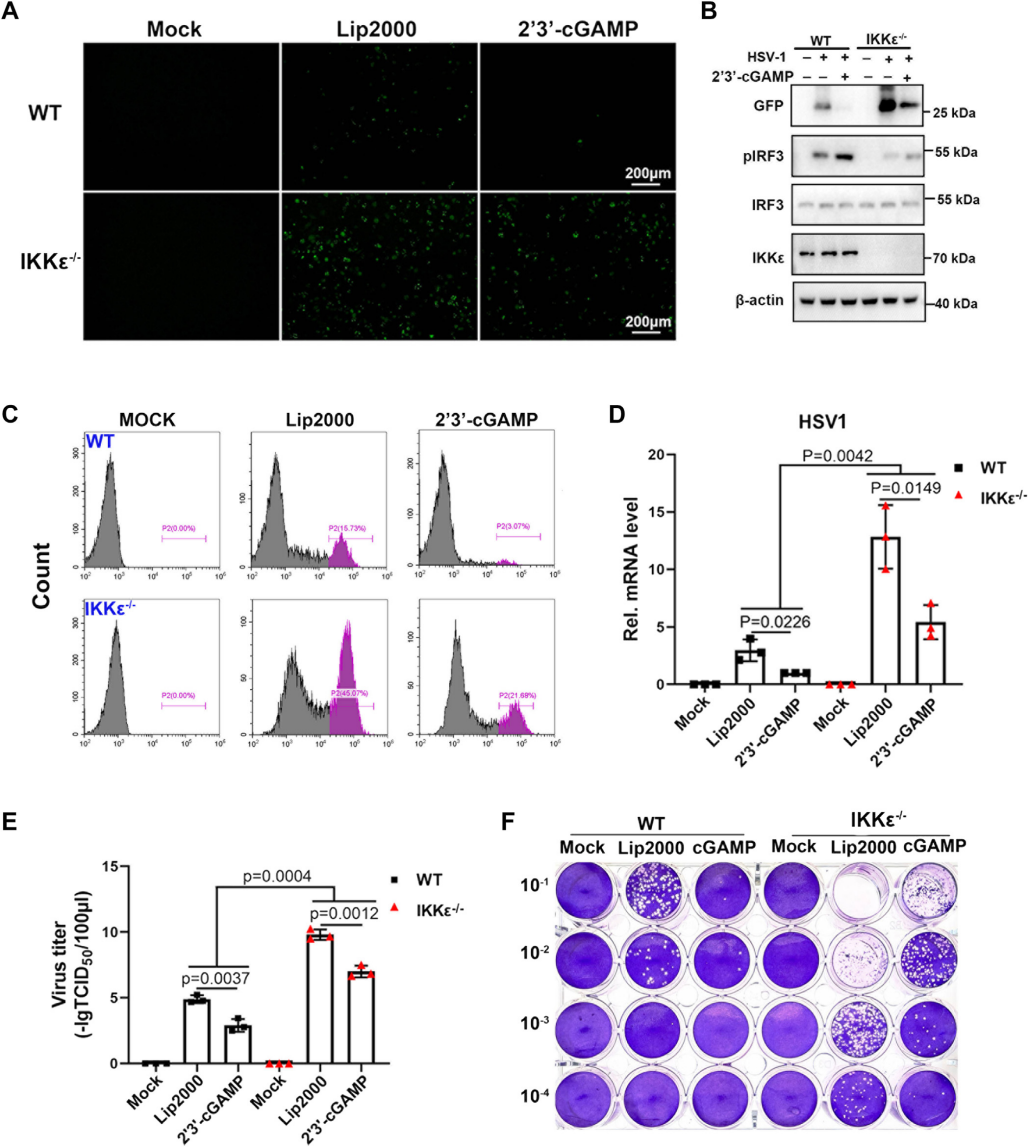
**pIKKε deficiency impairs Psting-mediated antiviral response.***A*–*D*, pIKKε^−/−^ 3D4/21 cells and 3D4/21 cells in 24-well plates (3 × 10^5^ cells/well) were stimulated with transfection of 2′3′-cGAMP (2 μg/ml) for 8 h. The stimulated cells were infected with HSV1-GFP (0.01 MOI) for 36 h. The GFP signals were visualized by fluorescence microscopy (*A*). The infected cells were harvested to measure the protein expressions by Western blotting (*B*), GFP signals by flow cytometry (*C*), and the HSV1 gB gene expression by RT–qPCR (*D*). *E* and *F*, the viral titer in the supernatant from HSV1-infected cells was measured by TCID_50_ assay (*E*) and plaque assay (*F*), respectively. HSV1, herpes simplex virus 1; MOI, multiplicity of infection; pIKKε, porcine IKKε; qPCR, quantitative PCR; STING, stimulator of interferon genes; TCID_50_, 50% tissue culture infectious dose.

To investigate whether IKKε participates in STING-mediated antiviral response, we selected a GFP virus herpes simplex virus 1 (HSV1, a DNA virus) to infect pIKKε^−/−^ and WT 3D4/21 cells with or without 2′3′-cGAMP treatment. Based on viral GFP signal, we observed the upregulated HSV1 replication in pIKKε^−/−^ cells as evidenced by fluorescence microscopy, Western blotting, and flow cytometry analysis (Fig 2, *A*–*C*). Consistently, we also found that phosphorylation of IRF3 was markedly decreased in pIKKε^−/−^ cells as compared with WT cells (Fig. 2*B*). Furthermore, the upregulation of HSV1 replication in pIKKε^−/−^ cells was shown by RT–quantitative PCR (qPCR) detection of viral gB gene transcription (Fig. 2*D*). Accordingly, the virus titers in the supernatants of pIKKε^−/−^ cells infected with HSV1 were increased relative to the WT cells in both 50% tissue culture infectious dose (TCID_50_) and plaque assays (Fig. 2, *E* and *F*). It should be noted that 2′3′-cGAMP exerts anti-HSV1 activity not only in WT 3D4/21 cells but also in pIKKε^−/−^ cells in aforementioned different assays, suggesting functional STING–TBK1 axis in the absence of IKKε in porcine macrophages. Collectively, these data proved that pIKKε participates in the pSTING-mediated antiviral activity.

### pIKKε interacts directly with pSTING on the CTT region

**Figure 3:**
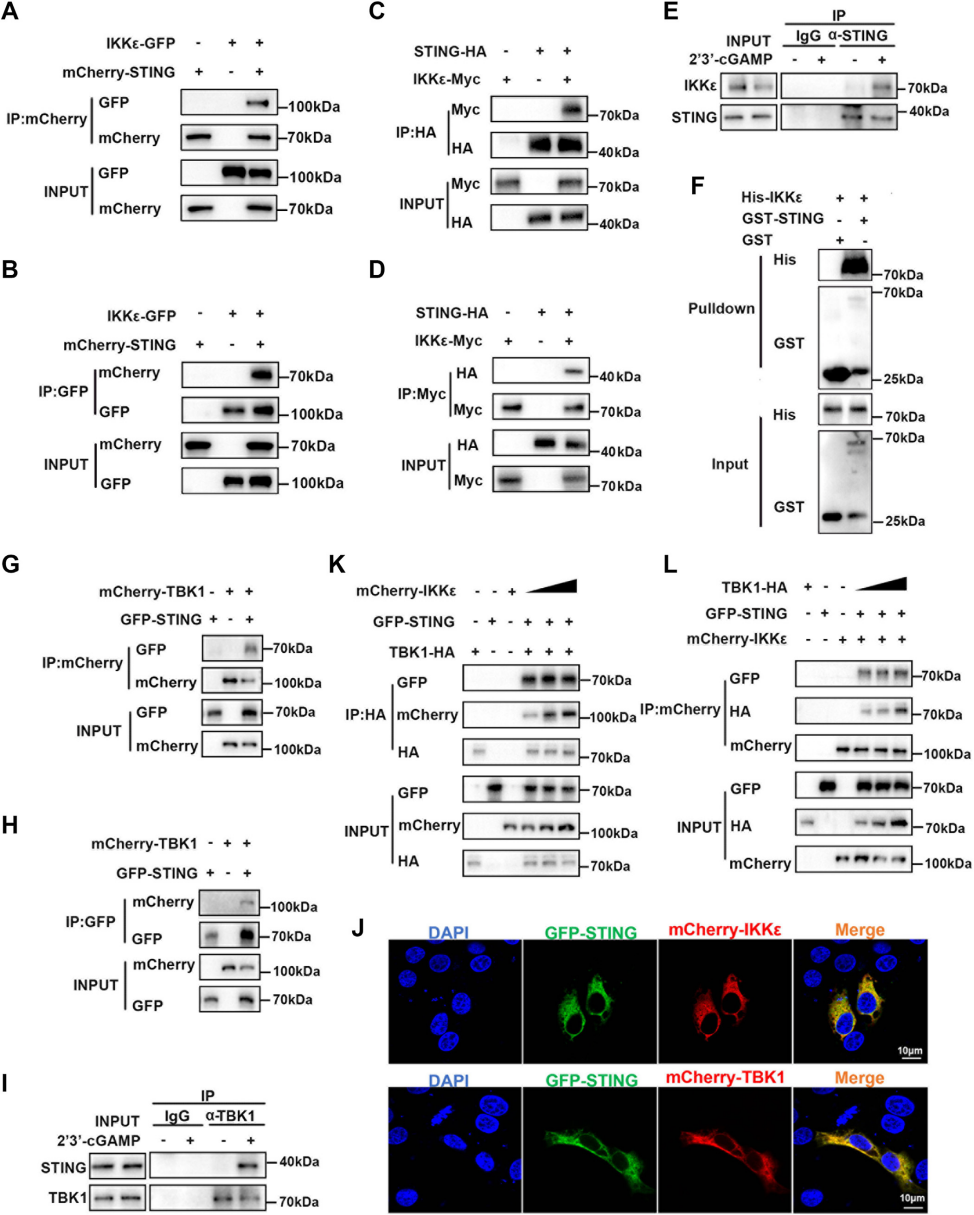
**pIKKε directly interacts with pSTING.***A*–*D*, HEK293T cells were cotransfected with pIKKε-GFP and mCherry-pSTING (*A* and *B*), with pIKKε-Myc and pSTING-HA (*C* and *D*) for 48 h. The cells were harvested and subjected for co-IP and subsequent Western blot analysis. *E*, 3D4/21 cells were stimulated with or without 2′3′-cGAMP (2 μg/ml), and then endogenous interaction between pIKKε and pSTING was identified by co-IP using anti-STING antibody. *F*, the purified GST-pSTING and His-pIKKε were incubated and subjected to GST pulldown with Glutathione Sepharose 4B resin, followed by Western blot analysis. *G* and *H*, HEK293T cells were transfected with mCherry-pTBK1 and GFP-pSTING for 48 h, followed by co-IP and Western blot analysis. *I*, 3D4/21 cells were stimulated with or without 2′3′-cGAMP (2 μg/ml), and then endogenous interaction between pSTING and pTBK1 was examined by co-IP using the TBK1 antibody. *J*, 3D4/21 cells in 24-well plates were cotransfected with mCherry-pIKKε/pTBK1 and GFP-pSTING for 24 h. Cells were fixed, stained, and examined for colocalization by confocal microscopy. *K* and *L*, 293T cells were cotransfected with 0.5 μg GFP-STING, 0.5 μg TBK1-HA, and increasing amounts of pIKKε (0.5, 1, and 1.5 μg) for 48 h (*K*), with 0.5 μg GFP-STING, 0.5 μg mCherry-IKKε and increasing amounts of pTBK1 (0.5, 1, and 1.5 μg) for 48 h (*L*). The cells were harvested and immunoprecipitated with anti-HA antibody and anti-mCherry antibody, respectively, followed by Western blot analysis. co-IP, coimmunoprecipitation; GST, glutathione-*S*-transferase; HEK293T, human embryonic kidney 93T cell line; pIKKε, porcine IKKε; STING, stimulator of interferon genes; TBK1, TANK-binding kinase 1.

It is well known that STING as an ER adaptor binds with TBK1 to activate the IRF3 and type I IFN response (9, 10); however, whether STING interacts with IKKε remain unknown. To identify the interaction between pSTING and pIKKε, several experiments were performed. Both the coimmunoprecipitation (co-IP) and reverse co-IP showed that exogenous pSTING interacts with pIKKε with different protein tags (Fig. 3, *A*–*D*). Furthermore, endogenous pSTING is also immunoprecipitated with pIKKε, which is very obvious upon cGAMP stimulation (Fig. 3*E*). In addition, the purified pSTING also directly interacts with the purified pIKKε in glutathione-*S*-transferase (GST) pull-down assay (Fig. 3*F*). As the control, the exogenous pSTING interacts with pTBK1 in both co-IP and reverse co-IP assay (Fig. 3, *G* and *H*). Endogenous pSTING is also immunoprecipitated with pTBK1 upon cGAMP stimulation (Fig. 3*I*). Complementary with these results, the pSTING-GFP was colocalized very well with IKKε-mCherry as well as TBK1-mCherry in the cytoplasm of transfected cells (Fig. 3*J*). Notably, the interactions between STING and IKKε and between STING and TBK1 were not disturbed by increasing amounts of TBK1 and IKKε, respectively (Fig. 3, *K* and *L*). Taken together, all these data demonstrated that pIKKε actually directly interacts with pSTING, which is similar to pTBK1.

**Figure 4:**
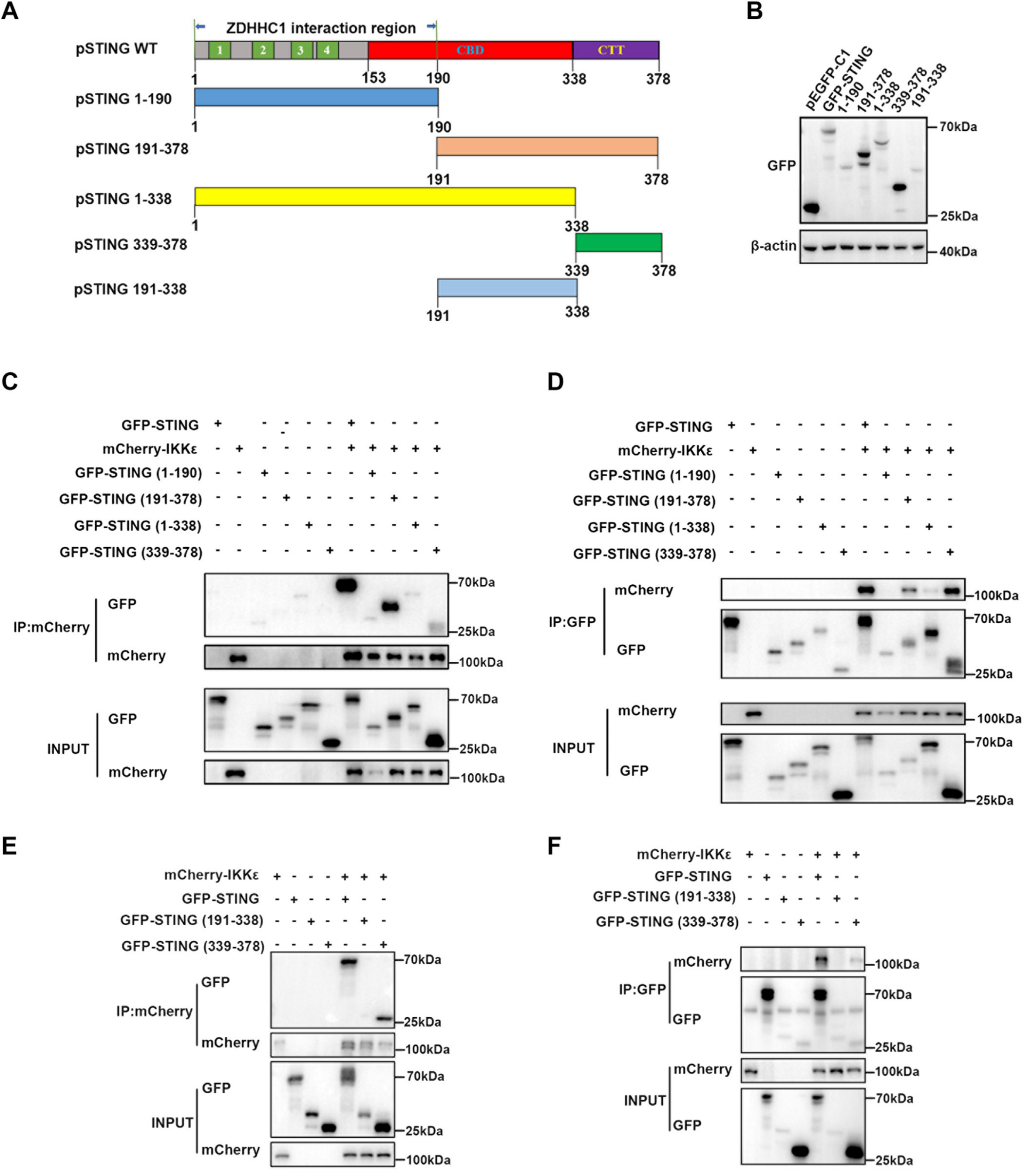
**The pIKKε interacts with pSTING on the C-terminal tail region.***A*, schematic of pSTING molecular structure and its truncated mutants. *B*, the protein expressions of full-length pSTING and its truncated mutants. *C*–*F*, each GFP-pSTING or its truncation mutants were cotransfected with mCherry-pIKKε into HEK293T for 48 h, and the cells were harvested and subjected for co-IP and subsequent Western blot analysis by using indicated antibodies. CBD, cyclic dinucleotide–binding domain; co-IP, coimmunoprecipitation; CTT, C-terminal tail; HEK293T, human embryonic kidney 293T cell line; pIKKε, porcine IKKε; STING, stimulator of interferon genes.

To identify which domain of pSTING is important for pSTING interaction with pIKKε, GFP-tagged pSTING and five truncated mutants were prepared, and their expressions were confirmed by Western blotting (Fig 4, *A* and *B*). Subsequently, co-IP and reverse co-IP assays were used to identify the pSTING domains important for pIKKε interaction. Domain mapping revealed that pIKKε interacts with pSTING (191–378 amino acids) and pSTING CTT (339–378 amino acids) but not with pSTING (1–190 amino acids) and pSTING (1–338 amino acids) (Fig. 4, *C* and *D*). Furthermore, the pIKKε does not interact with pSTING (191–338 amino acids) in both co-IP and reverse co-IP assays (Fig. 4, *E* and *F*). Based on these results, we inferred that the CTT domain of pSTING is necessary and sufficient for pSTING interaction with pIKKε.

### The CTT TBK1-binding motif (TBM) and leucine 373 residue within are critical for the interaction of pSTING with pIKKε

**Figure 5:**
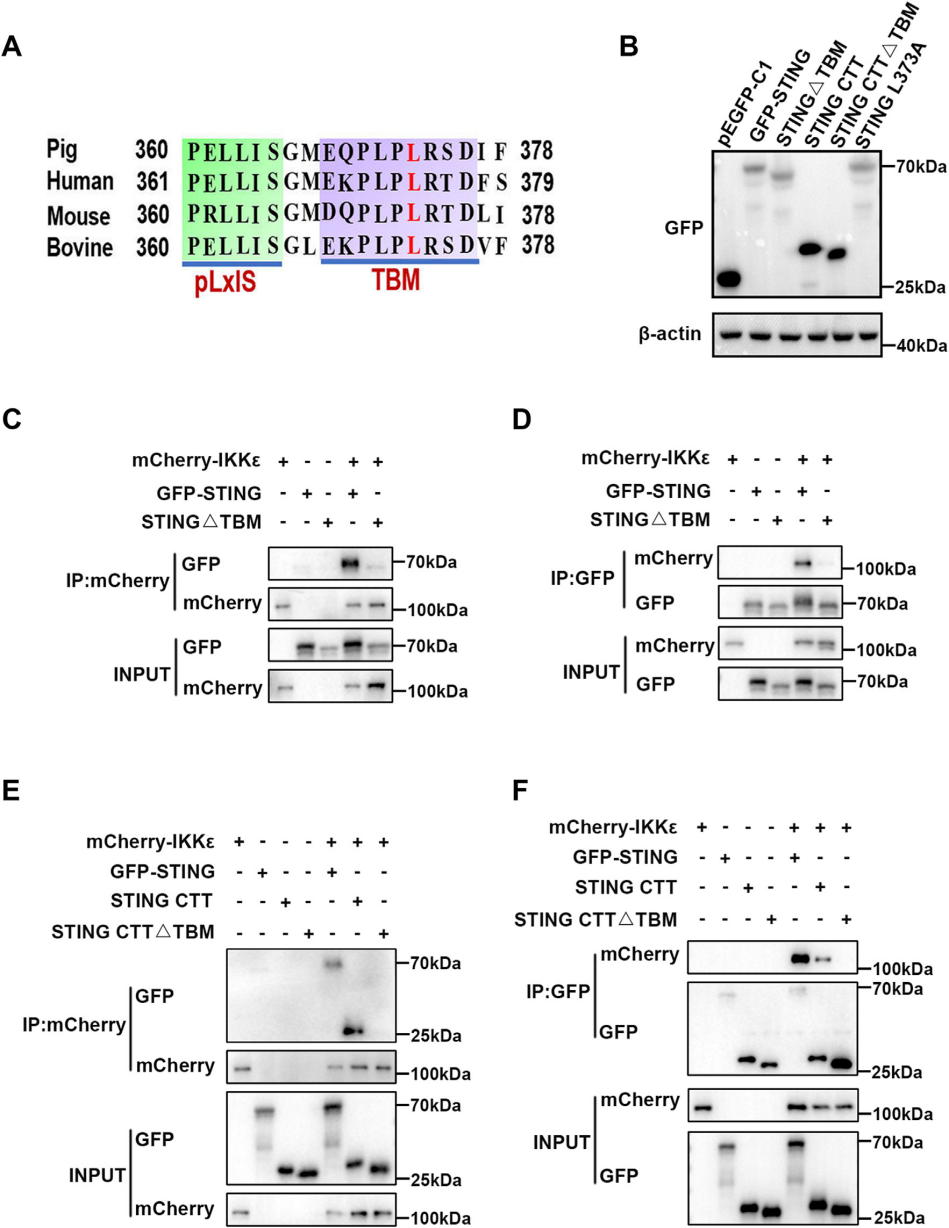
**The TBK1-binding motif is the key for the interaction between pSTING and pIKKε.***A*, sequence alignment of the C-terminal tail (CTT) of STING from porcine, human, mouse, and bovine. *B*, the protein expressions of full-length pSTING, pSTING CTT, and their mutants. *C* and *D*, the GFP-pSTING or pSTING△TBM was cotransfected with mCherry-pIKKε into HEK293T cells for 48 h, followed by co-IP and Western blot analysis. *E* and *F*, the GFP-pSTING, pSTING CTT, or pSTING CTT△TBM was cotransfected with mCherry-pIKKε into HEK293T for 48 h, followed by co-IP and subsequent Western blot analysis. co-IP, coimmunoprecipitation; HEK293T, human embryonic kidney 293T cell line; pIKK, porcine IKK; STING, stimulator of interferon genes; TBK1, TANK-binding kinase 1.

Previous studies including ours showed that the CTT of STING contains both TBK1 and IRF3 recruitment and activation motifs, which are essential for the induction of type I IFNs (9, 23). Considering TBM within the CTT of STING mediates its interactions with TBK1, we assumed that TBM is possibly a key domain for the interaction between pSTING and pIKKε. To verify this hypothesis, we built TBM deletion mutants in the contexts of both pSTING-GFP and pSTING CTT-GFP (Fig 5, *A* and *B*). The co-IP and reverse co-IP assays showed that pSTING ΔTBM cannot interact with pIKKε in transfected cells (Fig. 5, *C* and *D*). Meanwhile, it was found that the pSTING CTTΔTBM also loses the interaction with pIKKε (Fig. 5, *E* and *F*). These results implied that TBM within the CTT of pSTING is the key for the binding of pIKKε.

**Figure 6:**
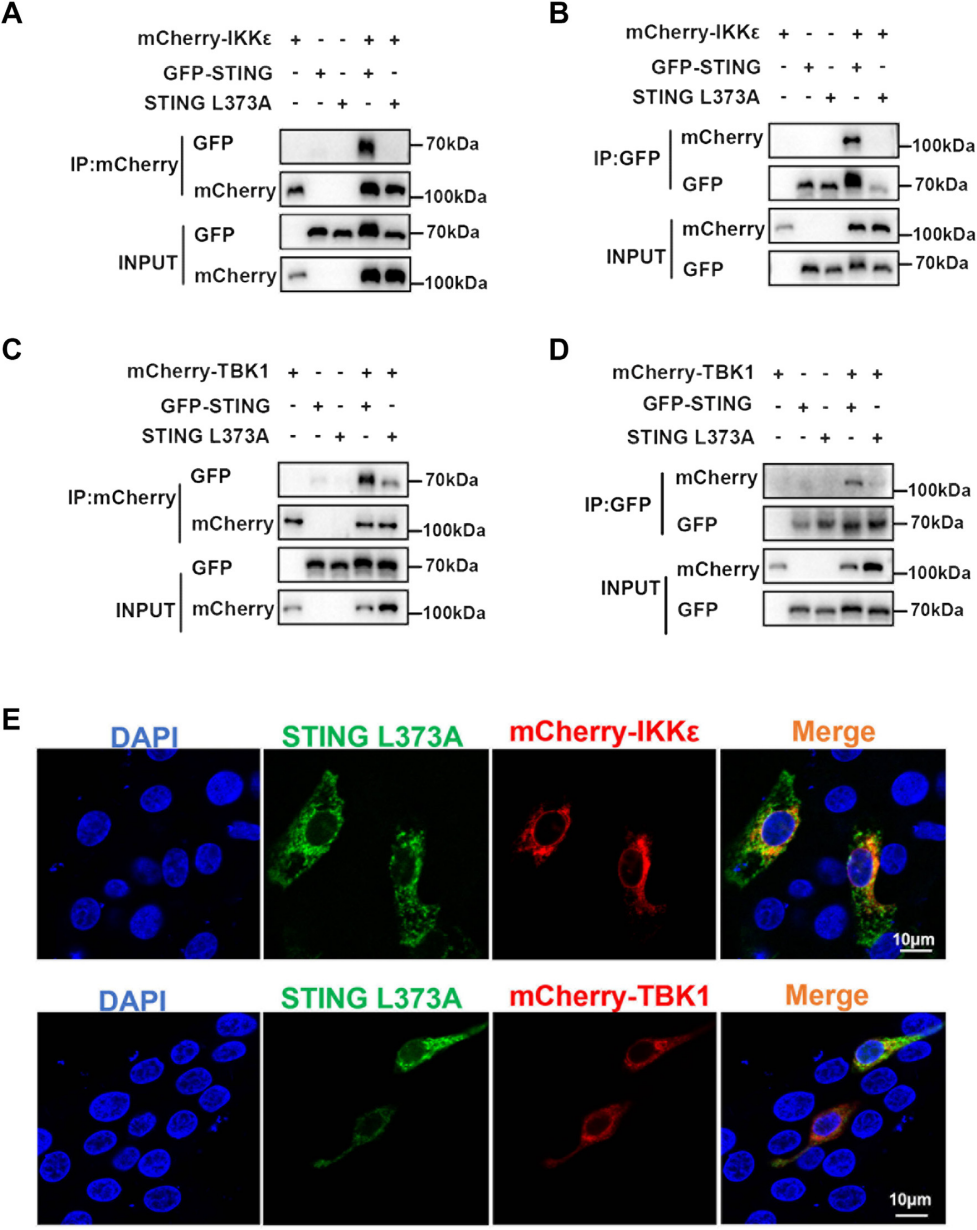
**The leucine 373 of pSTING is critical for its interaction with pIKKε and pTBK1.***A* and *B*, GFP-pSTING or pSTING L373A was cotransfected with mCherry-pIKKε into HEK293T for 48 h, followed by co-IP and subsequent Western blot analysis. *C* and *D*, GFP-pSTING or pSTING L373A was cotransfected with mCherry-pTBK1 into HEK293T for 48 h, followed by co-IP and subsequent Western blot analysis. *E*, 3D4/21 cells in 24-well plates were cotransfected with GFP-pSTING L373A and mCherry-pIKKε/pTBK1-mCherry for 24 h. Cells were fixed, stained, and examined for cellular colocalization by confocal microscopy. co-IP, coimmunoprecipitation; HEK293T, human embryonic kidney 293T cell line; pIKKε, porcine IKKε; pTBK1, phosphorylated TANK-binding kinase 1; STING, stimulator of interferon genes.

Previous study reported that human STING leucine 374 within TBM (equivalent to pSTING L373) is important for TBK1 binding to STING, and L374A mutation abolishes the STING interaction with TBK1 (10). To identify whether leucine L373 is required for pSTING interaction with pIKKε, we made the pSTING L373A mutant (Fig. 5, *A* and *B*) and examined its interaction with both pIKKε and pTBK1. Intriguingly, there was no observed interaction between pSTING L373A and pIKKε in co-IP and reverse co-IP assays (Fig 6, *A* and *B*). Besides, the interaction between pSTING L373A and pTBK1 largely disappeared (Fig. 6, *C* and *D*), which was as expected. Consistently, there was no colocalization between pSTING L373A and pTBK1 or pIKKε in transfected cells (Fig. 6*E*). These results suggested that leucine 373 residue within TBM is important for pSTING binding to pIKKε.

### Both kinase domain and scaffold dimerization domain of IKKε participate in the interaction with pSTING/CTT and the antiviral function

**Figure 7:**
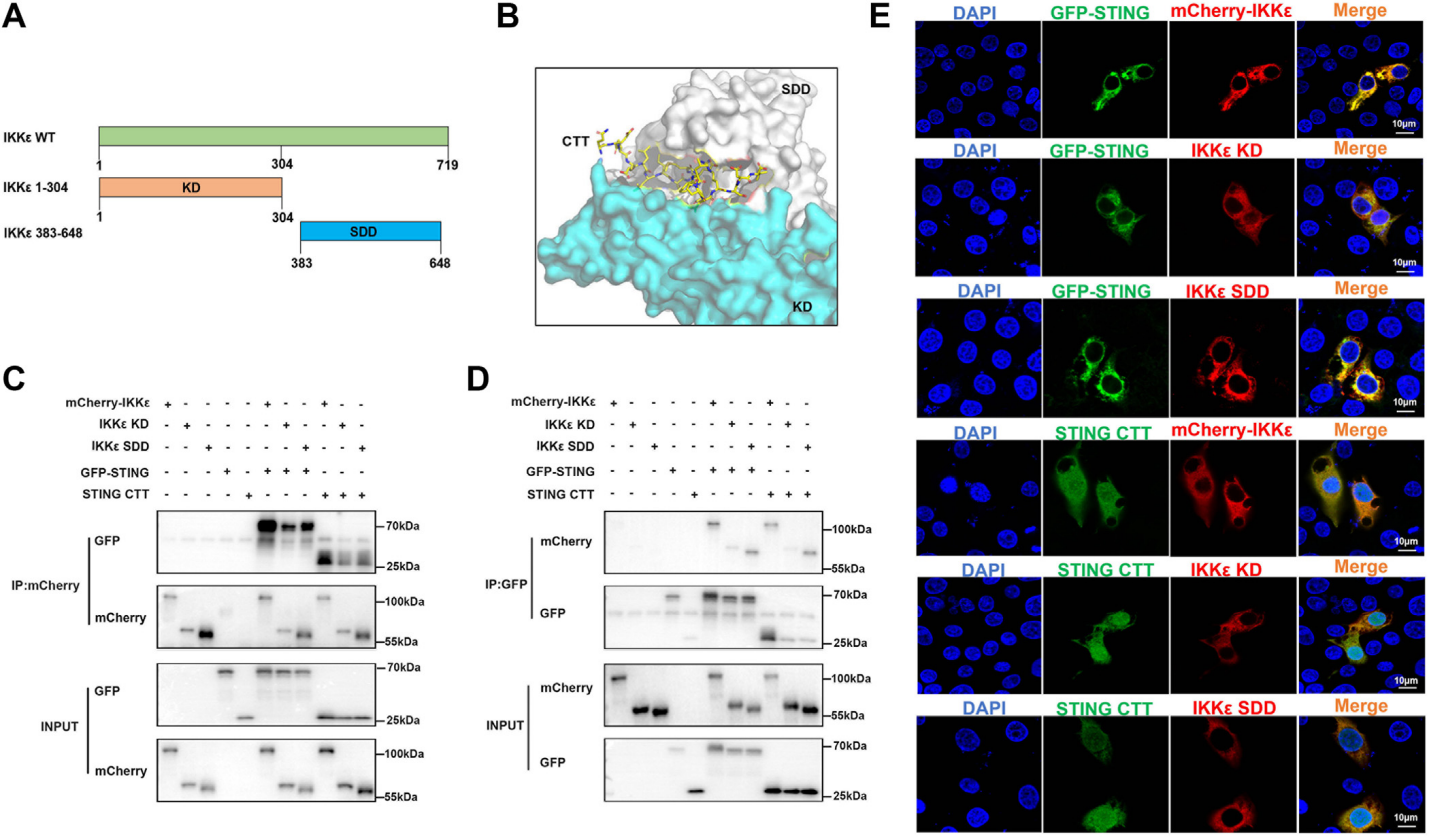
**The pSTING and CTT interact with KD and SDD of pIKKε.***A*, schematic of full-length pIKKε and its truncated mutants. *B*, structural view of the binding interface between pIKKε and pSTING CTT. *C* and *D*, GFP-pSTING or pSTING CTT was cotransfected with pIKKε truncation mutants into HEK293T for 48 h, and the cells were harvested and subjected for co-IP using subsequent Western blot analysis. *E*, each mCherry-pIKKε mutant was cotransfected with STING-GFP or STING CTT into 3D4/21 cells for 24 h, and then the cells were fixed, stained, and examined for cellular colocalization by confocal microscopy. co-IP, coimmunoprecipitation; CTT, C-terminal tail; HEK293T, human embryonic kidney 293T cell line; KD, kinase domain; pIKKε, porcine IKKε; SDD, scaffold dimerization domain; STING, stimulator of interferon genes.

IKKε is comprised of two major domains: the N-terminal kinase domain (KD) and the C-terminal scaffold dimerization domain (SDD) (Fig. 7*A*) (24, 25). Because SDD of pIKKε interacts with tumor necrosis factor receptor–associated factor 3, whereas KD interacts with IRF3 (26), it is possible that pSTING/CTT binds to the KD and/or SDD of IKKε. To verify the hypothesis, molecular modeling was first applied to show the binding interface between pIKKε and porcine STING/CTT where both KD and SDD are involved in binding with CTT (Fig. 7*B*). The co-IP and reverse co-IP assays were performed in cells cotransfected with GFP-pSTING/CTT plus mCherry-tagged pIKKε and its KD and SDD expression plasmids. The results showed that pSTING and CTT not only immunoprecipitated with pIKKε and its SDD but also weakly with KD (Fig. 7, *C* and *D*). Confocal microscopy analysis also revealed that pSTING and CTT were obviously colocalized with pIKKε and SDD and were weakly but clearly colocalized with KD (Fig. 7*E*). All results implicated that pSTING/CTT interacts with both KD and SDD of pIKKε.

**Figure 8:**
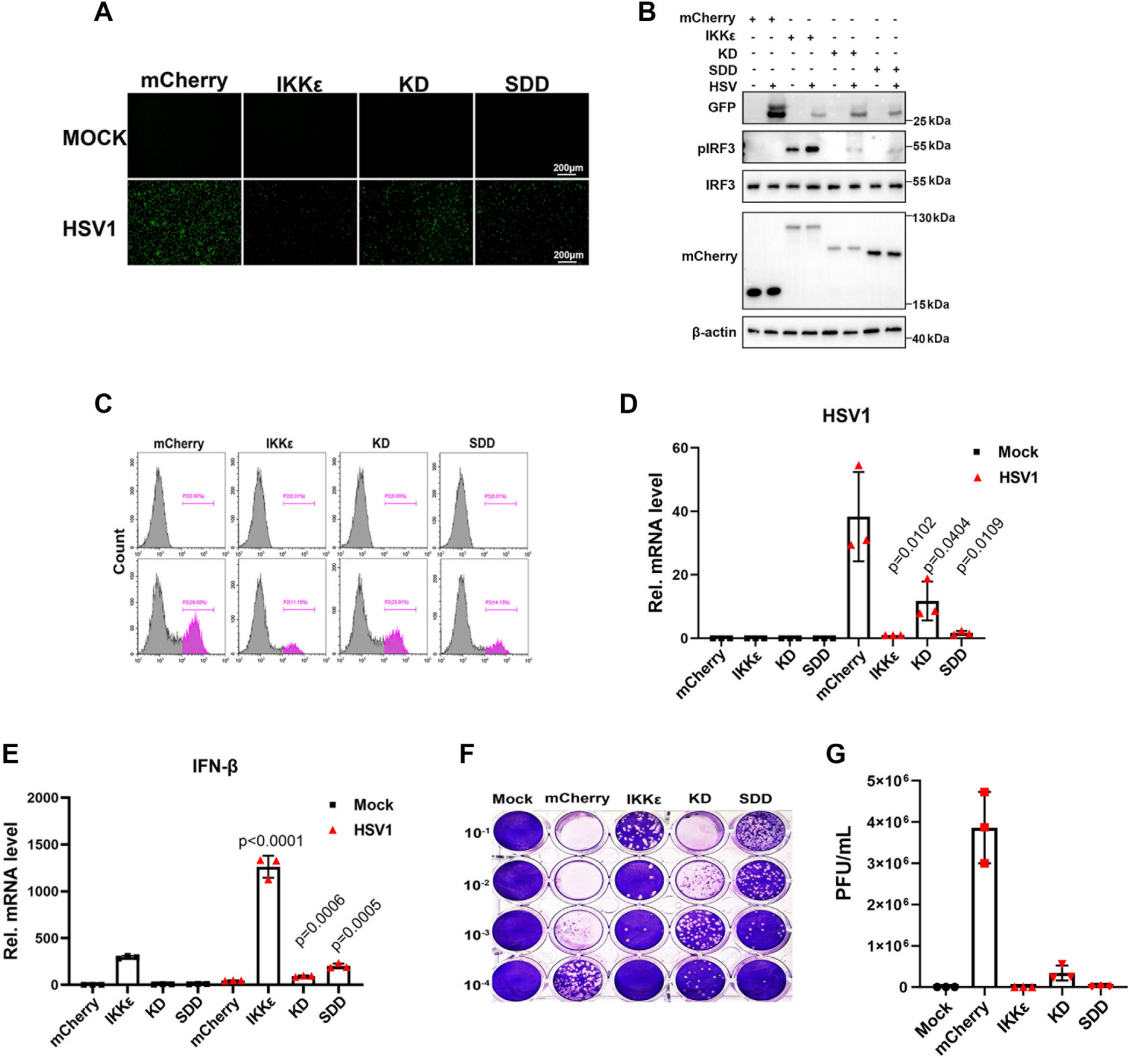
**Reconstitution of pIKKε in IKKε**^**−/−**^**cells confirms pIKKε participation in STING-mediated antiviral response.***A*–*E*, pIKKε^−/−^ 3D4/21 cells in 24-well plates were transfected with IKKε or pIKKε mutants (1 μg each) for 24 h and then infected with 0.01 MOI HSV1 for 24 h. The GFP signals were visualized by fluorescence microscopy (*A*). The infected cells were harvested to measure the protein expressions by Western blotting (*B*), GFP signals by flow cytometry (*C*), and the HSV1 gB gene and host IFNβ gene expressions by RT–qPCR (*D* and *E*). *F* and *G*, the viral titer in the supernatant from HSV1-infected cells was measured by plaque assay (*F*), and the number of plaques are plotted in (*G*). HSV1, herpes simplex virus 1; IFN, interferon; MOI, multiplicity of infection; pIKKε, porcine IKKε; qPCR, quantitative PCR; STING, stimulator of IFN genes.

To further validate the aforementioned results and the antiviral function of pIKKε, we transfected the pIKKε and its two mutants in IKKε^−/−^ cells, and the reconstituted cells were infected with HSV1. The HSV1 replication was examined, and the results showed that, relative to vector control, reconstitution of pIKKε, SDD, and KD all exhibited significant antiviral activity as evidenced by fluorescence microscopy (Fig. 8*A*), Western blotting (Fig. 8*B*), flow cytometry (Fig. 8*C*), RT–qPCR (Fig. 8*D*), and plaque assay (Fig. 8, *F* and *G*). Generally, the KD mutant possessed less potent anti-HSV1 activity than SDD and pIKKε, which is in accordance with its lower ability to induce IRF3 phosphorylation (Fig. 8*B*) and IFNβ gene transcription (Fig. 8*E*). Hence, these data confirmed that both SDD and KD of pIKKε are involved in the pSTING-mediated antiviral activity.

## Discussion

Although it has been widely believed that TBK1 alone mediates STING-induced type I IFN expression and NF-κB response (9, 10, 11, 14, 27), the concept might be dependent on cell type, species, or other parameters. The early work was performed on TBK^−/−^ mouse embryonic fibroblasts that exhibited abrogated IRF3 and NF-κB p65 phosphorylation upon dsDNA90 stimulation, whereas the treatment of mouse embryonic fibroblasts with IKKε siRNA had little effect on downstream IRF3 and p65 phosphorylation (14). Using the *in vitro* reconstitution system and mouse L929 cells, TBK1 was shown to mediate STING-triggered IRF3 activation (11). Later on, the crystal structural dissection of STING and TBK1 complex further strengthened the sole role of TBK1 in STING activation and signaling (9, 10). However, a recent study revealed that TBK1 and IKKε act redundantly to mediate STING-induced NF-κB response in mouse myeloid cells and *in vivo*, despite that TBK1 is still predominantly required for IRF3 activation and IFN response (20). Another recent study demonstrated that IKKϵ expression can compensate for TBK1 to ensure the efficient type I IFN response in human myeloid cells (21).

IKKε has been shown to transmit the cascade signals of other adaptor proteins, such as mitochondrial antiviral signaling protein for RIG-1-like receptors (25, 28, 29). Here, we come up with evidences that in addition to pTBK1, pIKKε has also great importance for pSTING-mediated type I IFN expression and antiviral function in porcine macrophages (Figs. 1 and 2). Our results also suggested that pIKKε cooperates with pTBK1 to mediate pSTING-elicited type I IFN production, whereas pSTING-induced NF-κB response is mediated by a pTBK1 and pIKKε redundant mechanism (Fig. 1). Thus, we conclude that pIKKε function as a vital positive upstream kinase in STING signaling triggered IRF3 phosphorylation and downstream antiviral type I IFN response. Our conclusion is supported not only by the high degree of similarity between IKKε and TBK1 (17, 18) but also by the immune evasion of IKKε by different viruses including some DNA viruses (22, 24, 30, 31).

**Supporting Figure S1:**
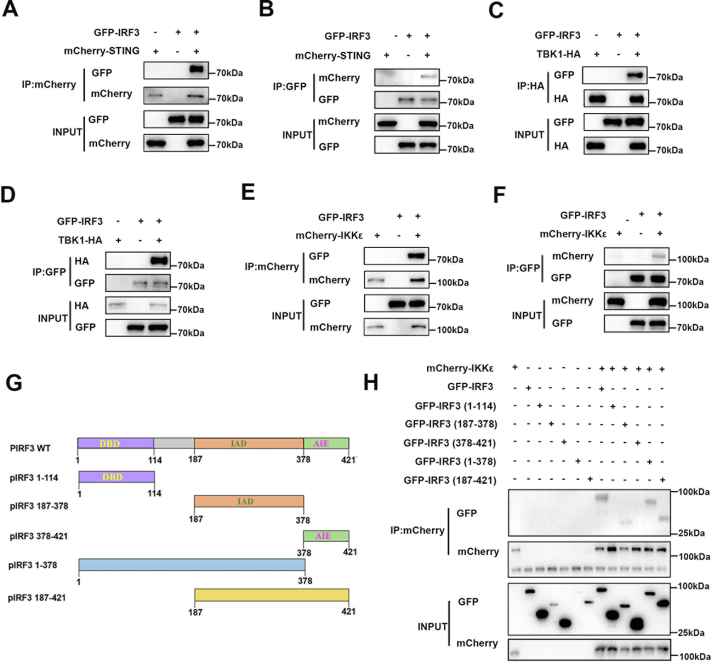
**The interactions of pSTING/pTBK1/pIKKε with pIRF3.***A*–*F*, HEK293T cells were co-transfected with the indicated plasmids of GFP-pIRF3 together with mCherry-pSTING, pTBK1-HA or mCherry-pIKKε for 48 h. Cells were harvested and subjected for Co-IP and subsequent Western blot analysis using the indicated antibodies. *G*, Schematic of full-length pIRF3 and its truncated mutants. DBD, DNA binding domain; IAD, IRF association domain; AIE, auto-inhibition element. *H*, HEK293T cells were co-transfected with mCherry-pIKKε and each GFP-pIRF3 truncation mutants for 48 h and then the cells were harvested and subjected for Co-IP and subsequent Western blot analysis using the indicated antibodies.

Confirming the important role of TBK1 in STING-mediated IRF3 and NF-κB responses, our results showed that pTBK1 interacts with pSTING (Fig. 3, *E*–*H*). Analogously, pIKKε also interacts directly with pSTING (Fig. 3, *A*–*D*). Besides, we also showed that pIKKε interacts with pSTING on the CTT (Fig. 4), which was shown to be sufficient and necessary to activate TBK1 and stimulate IRF3 phosphorylation *in vitro* reconstitution system (11). STING CTT contains a pLxIS motif and a TBM, which are conserved and important for IRF3 and TBK1 binding and recruitment, respectively (9, 32, 33). We showed that TBM of pSTING is the key for binding with pIKKε (Fig. 5). Furthermore, the leucine (L) residue 373 within TBM, which is specifically important for TBK1 binding (9, 10), was also critical for pIKKε binding (Fig. 6). Consistent with TBK1 (9, 10), both SDD and KD of pIKKε participate in the interaction with pSTING CTT and the downstream antiviral function (Figs. 7 and 8). On the other hand, pIKKε, pTBK1, and pSTING all interact with pIRF3 (Fig. S1, *A*–*F*). Furthermore, pIKKε interacts with pIRF3 on the IRF3 association domain (187–378 amino acids) (Fig. S1, *G* and *H*), which was shown to be responsible for TBK1 interaction (34, 35). In one word, the pIKKε and pTBK1 interact with upstream pSTING and downstream pIRF3 in a totally identical way.

**Figure 9:**
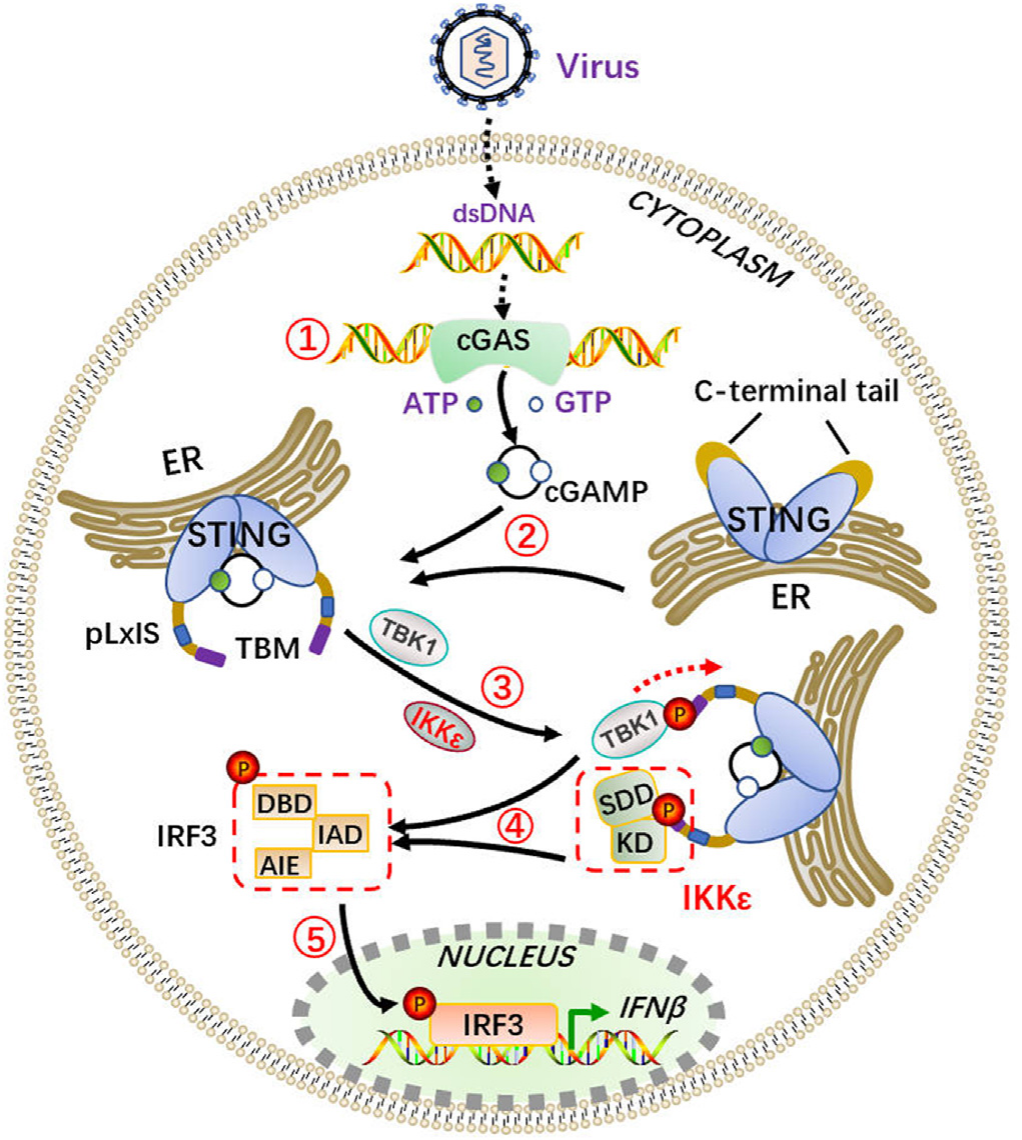
**The schematic diagram of pIKKε together with pTBK1 to mediate porcine cGAS–STING pathway–induced type I interferon response.** DNA virus infects cells and releases viral nucleic acid into cells. ① cGAS recognizes and binds with the dsDNA, and the activated cGAS utilizes GTP and ATP to synthesize 2′3′-cGAMP. ② 2′3′-cGAMP, as a second messenger, binds with STING and causes its oligomerization and translocation from ER. ③ The oligomerized STING recruits TBK1 as well as IKKε *via* the conserved TBK1-binding motif (TBM) of C-terminal tail (CTT). ④ Both TBK1 and IKKε are activated and phosphorylate the Ser365 in the conserved pLxIS motif of CTT, allowing recruiting IRF3 on its IRF3 association domain (IAD). ⑤ The phosphorylated IRF3 translocates into cell nucleus to induce the production of type I IFNs. cGAS, cyclic GMP–AMP synthase; ER, endoplasmic reticulum; IFN, interferon; pIKKε, porcine IKKε; pTBK1, phosphorylated TANK-binding kinase 1; STING, stimulator of interferon genes.

Since pIKKε–pTBK1 interact with pSTING and pIRF3 on the same sites, it is possible that competitive relationship may exist between pIKKε and pTBK1. However, we did not observe such a competition from either pIKKε^−/−^ or pTBK1^−/−^ cells; instead, pIKKε and pTBK1 are cooperative (Fig. 1). Consistently, both pIKKε and pTBK1 exist with STING in one complex, the increasing amounts of pIKKε do not disturb the interaction of pTBK1 and pSTING, and vice versa (Fig. 3, *K* and *L*). Based on all our results, we propose the following model: upon activation, pSTING recruits both pTBK1 and pIKKε during translocation from ER to Golgi apparatus. The pSTING, pTBK, pIKKε, and pIRF3 may form a signalosome by which both pTBK1 and pIKKε phosphorylate pIRF3 and other substrates for NF-κB activation (Fig. 9). Since pIKKε and pTBK1 play similar function, why are both kinases required to achieve maximal activation of the type I IFN response? We reason that it may be a security mechanism for antiviral IFN response of host during biological evolution. As for how pIKKε and pTBK1 work exactly together for IFN response, both kinases are constitutively and constantly expressed in porcine macrophages with cGAMP stimulation (Figs. 1, *D*–*F* and 2*B*), it may be less likely the scenario where the early STING-induced IFN response is TBK1 predominant, whereas the late STING-induced IFN response is IKKε dependent. Nevertheless, it is interesting to be explored in the future.

In conclusion, our present study provides genetic and biochemical evidence to illustrate that pIKKε directly participates in regulating STING-mediated type I IFN expression and antiviral function by synergizing with pTBK1. These findings deepen our understanding of porcine cGAS–STIN–IFN antiviral signaling pathway and will be helpful for formulating antiviral strategies against porcine viral diseases.

## Experimental procedures

### Cells, virus, and reagents

Human embryonic kidney 293T and Vero cells were cultured in Dulbecco's modified Eagle's medium (DMEM) containing 10% fetal bovine serum (FBS), 100 IU/ml of penicillin, and 100 μg/ml streptomycin. Porcine alveolar macrophages (3D4/21) were cultured in RPMI1640 medium supplemented with 10% FBS and 100 IU/ml of penicillin plus 100 μg/ml streptomycin. All cells were grown at 37 °C with 5% CO_2_. The HSV1 (HSV1-GFP) has been kept and used in our laboratory (36). TRIpure RNA Reagent was purchased from Aidlab. HiScript first Strand complementary DNA (cDNA) Synthesis Kit, ChamQ Universal SYBR qPCR Master Mix, and 2× Taq Master Mix (Dye plus) were all from Vazyme Biotech Co, Ltd, Restriction endonucleases BglII, KpnI, EcoRI, EcoRV, BbsI, SalI, and DnpI were purchased from New England Biolabs. Lipofectamine 2000 was acquired from ThermoFisher Scientific. The STING agonist 2′3′-cGAMP was bought from InvivoGen. Protein A/G Plus-Agarose was acquired from Santa Cruz Biotechnology. The 4′,6-diamidino-2-phenylindole was from Beyotime Biotech.

### Antibodies

Mouse antiactin monoclonal antibody (mAb), mouse anti-GFP mAb, mouse anti-HA mAb, and mouse anti-His mAb were all from Transgen Biotech. The mouse anti-GST mAb was bought from Abmart Biotech. The rabbit anti-TBK1 (D1B4), anti–phospho-TBK1 (D52C2), anti-IRF3 (D614C), anti-IκBα (44D4), and anti-HA (C29F4) mAbs were bought from Cell Signaling Technology. The rabbit anti–phospho-IRF3 mAb and anti-IKKε mAb were acquired from ThermoFisher Scientific and The HUABIO, respectively. Rabbit anti-STING pAb, mouse anti-mCherry, and anti-Myc mAbs were purchased from ProteinTech. The rabbit anti-ISG56-pAb was homemade in our laboratory. The horseradish peroxidase antimouse immunoglobulin G and horseradish peroxidase anti-rabbit immunoglobulin G were purchased from Sangon Biotech.

### Plasmid construction and gene mutation

The cDNA corresponding to the pIKKε gene (GenBank: XM_021063311) was amplified by RT–PCR using total RNA extracted from 3D4/21 cells and then cloned into the EcoRI/EcoRV sites of pCAGGS-Myc vector to produce pIKKε-Myc and the BglII/KpnI sites of pEGFP-N1 and pmCherry-C1 vectors to make pIKKε-GFP and mCherry-pIKKε, respectively. The KD and SDD of IKKε were amplified and subcloned into pmCherry-C1, designed as mCherry-KD and mCherry-SDD, respectively. The pTBK1 gene (GenBank: XM_021090852.1) amplified by RT–PCR from cDNA was cloned into the BglII/KpnI sites of pmCherry-C1 vector and EcoRI/EcoRV sites of pCAGGS-HA vector, to produce mCherry-pTBK1 and pTBK1-HA, respectively. The pSTING recombinant pEGFP-C1 and mCherry-C1 vectors were constructed in our previous study (37). The GFP-pSTING mutants, including pSTING (1–190 amino acids), pSTING (191–378 amino acids), pSTING (1–338 amin acids), and pSTING (339–378 amin acids) were made by subcloning in our previous study (30), whereas pSTING (191–338 amino acids), pSTING ΔTBM, pSTING CTT ΔTBM, and pSTING L373A were produced by mutation PCR from corresponding templates. The recombinant pSTING pcDNA-HA and pIRF3 pEGFP-C1 vectors were made in our previous study (23), whereas GFP-pIRF3 mutants, including pIRF3 (1–114 amino acids), pIRF3 (187–378 amino acids), pIRF3 (378–421 amino acids), pIRF3 (1–378 amin acids), and pIRF3 (187–421 amino acids) were PCR amplified and subcloned into the BglII/KpnI sites of pEGFP-C1 vector.

The pSTING gene was PCR amplified and cloned into the EcoRI/SalI sites of the prokaryotic pGEX-6p-1 vector. The pIKKε gene was first cloned into the SalI/EcoRV sites of the Gateway entry vector pENTR4-2HA. Next, the pIKKε-HA was transferred from pENTR4 vector to the Destination pDEST527 vector (Addgene) by LR recombination (ThermoFisher Scientific) to obtain the prokaryotic pIKKε expression plasmid.

The cloning ligation was performed with either T4 DNA Ligase (ThermoFisher Scientific) or 2×MultiF Seamless Assembly Mix was from Abclonal. The cloning PCR and mutation PCR were conducted with Golden Star T6 Super PCR mix polymerase (Tsingke) and KOD plus neo polymerase (Toyobo), respectively. All the cloning and mutation PCR primers were shown in Table S1.

### Quantitative real-time PCR

Total RNA was extracted from 3D4/21 in 24-well plates by using TRIpure reagent, and reverse transcription was performed using HiScript first Strand cDNA Synthesis Kit. The quantitative PCR was conducted with ChamQ Universal SYBR qPCR Master Mix. The qPCR program used was 95 °C for 30 s, followed by 40 cycles of 95 °C for 10 s and 60 °C for 30 s. The qPCR reactions were run on the StepOne Plus equipment (Applied Biosystems).The qPCR primers for pIFNβ, pISG56, pIL8, pβ-actin, HSV1 gB, and GFP are shown in Table S2.

### Gene knockout by CRISPR–Cas9 approach

**Supporting Figure S2:**
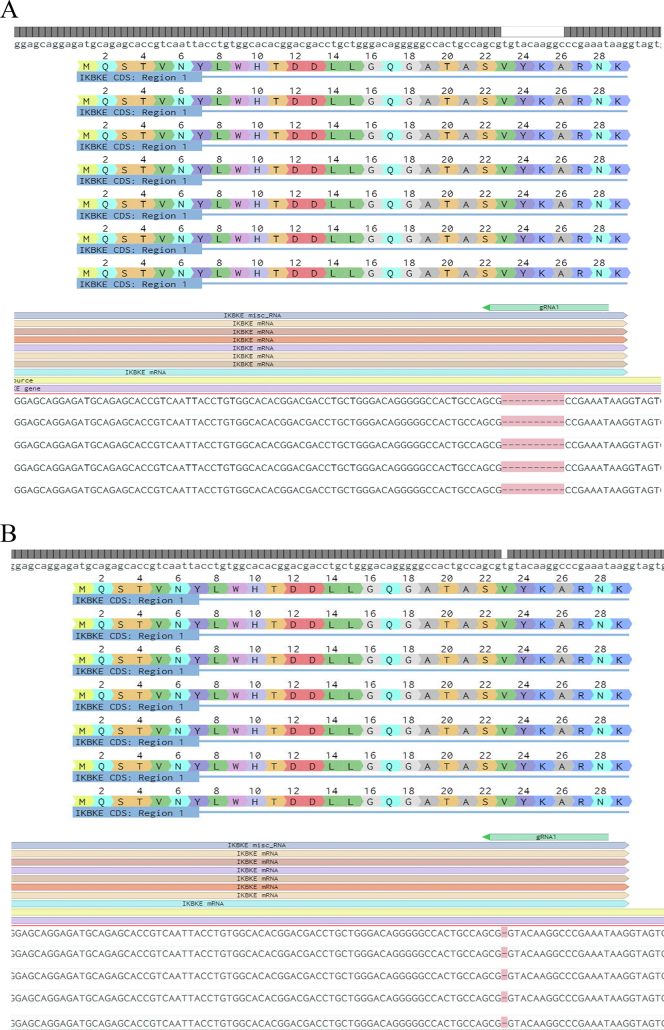
**The IKKε gene editing in 3D4/21 cell clones from CRISPR gRNA treatment.** The porcine IKKε genome DNA regions covering the gRNAs were amplified by PCR using the primers as shown in Table S3. The IKKε gene PCR products of individual 3D4/21 cell clones (*A*) and individual TBK1^−/−^ 3D4/21 cell clones (*B*) were ligated into T vectors and the inserted DNA fragments were sequenced for analysis of base insertion/deletion (indel) mutations. Shown are representatives of one IKKε^−/−^ cell clone (*A*) and one IKKε^−/−^TBK1^−/−^ cell clone (*B*) obtained and used in subsequent experiments.

The CRISPR guide RNA targeting pIKKε gene (GenBank: NC_010451; region: 67123055–67147991) was designed using the web tool from Benchling (www.benchling.com). The annealed guide RNA–encoding DNA sequences (Table S3) were cloned into BbsI site of pX458-EGFP. The recombinant vector was transfected into 3D4/21 cells or TBK1^−/−^ 3D4/21 cells (36) using Lipofectamine 2000 for 24 h. Then, the GFP-positive cells were sorted by flow cytometry into 96-well plates for monoclonal cell growth. The individual clones were detected for genomic DNA editing by PCR with primers shown in Table S3. The genomic PCR products were cloned into T vector using pClone007-versatile simple vector kit (TsingKe), and inserted fragments were multiply sequenced. The multiple sequenced genomic DNA fragments were compared with the template DNA to deduce the genomic editing, and thus the pIKKε^−/−^ cell clone and pIKKε^−/−^pTBK1^−/−^cell clone were obtained, respectively (Fig. S2). The pIKKε^−/−^ cells and pIKKε^−/−^pTBK1^−/−^cells were also examined for IKKε protein expression by Western blotting (Fig. 1, *D*–*F*).

### Flow cytometry

Briefly, 3D4/21 cells and 3D4/21 IKKε^−/−^ cells were stimulated or not by transfection with 2′3′-cGAMP for 8 h. After that, the cells were infected or not with HSV1-GFP (0.01 multiplicity of infection) for 24 h. After infection, the cells were harvested with trypsin digestion and washed three times with PBS. HSV1-GFP replication was analyzed by flow cytometry based on the GFP signal. In the constitution experiment, the IKKε^−/−^ cells were transfected with vector control or IKKε, or its mutants for 36 h, and then the cells were infected or not with HSV1-GFP (0.01 multiplicity of infection). After infection for 24 h, the cells were harvested and analyzed by flow cytometry.

### Viral TCID50 assay and plaque assay

The 10-fold serially diluted supernatants of virus-infected cells were used to infect Vero cells in 96-well plates for 2 h, with each dilution of eight replicates. Vero cells were washed and maintained in DMEM containing 2% FBS and grown at 37 °C for 3 to 4 days. The cytopathic effects were counted, and TCID_50_ values were calculated by Reed–Muench method.

Similarly, 10-fold serially diluted supernatants of virus-infected cells were used to infect Vero cells in 24-well plates for 2 h. Vero cells were overlaid by immobilizing medium of 1:1 mixture of warmed 2× DMEM with 4% FBS and a stock solution of heated 1.6% low melting agarose (Sigma–Aldrich). Four days postinfection, the immobilizing medium was discarded by tipping, and cells were fixed and stained with crystal violet cell colony staining solution for 1 h at room temperature. After washing with tap water, the clear plaques appeared and photos were taken.

### co-IP and Western blot analysis

Cells in 6-well plate were transfected with indicated plasmids for 24 h and lysed in lysis buffer (50 mM Tris [pH 7.2], 150 mM NaCl, 1% sodium deoxycholate, and 1% Triton X-100). The cell lysates were incubated with indicated antibodies overnight at 4 °C, and then protein A/G bead solution was added for another 2 h. Later, the beads were washed five times with cell lysis buffer and eluted with 2× SDS sample buffer. For Western blot analysis, the cell lysates and eluted protein samples were resolved with 6 to 10% SDS-polyacrylamide gel, and then transferred to a polyvinylidene fluoride membrane. After incubating with primary antibodies and secondary antibodies, protein signal on the membrane was visualized by Western blot imaging system (Tanon).

### GST pull-down assay

The prokaryotic GST-pSTING and His-pIKKε expression plasmids were transformed into *Escherichia coli* BL21/DE3, and the fusion proteins were induced by 0.5 mM IPTG at 25 °C for 9 h. The His-pIKKε was purified by His-tag Protein Purification Kit (Beyotime Biotech) from the supernatant of lysed bacteria. The expressed GST-pSTING and control GST proteins in the supernatant of lysed bacteria were bound with Glutathione Sepharose 4B resin (Solarbio). The GST protein–bound beads were next incubated with 1 μg purified His-pIKKε overnight at 4 °C, followed by elution and immunoblot analysis.

### Protein structure modeling and docking analysis

The three dimensional structures of pIKKε (GenBank: XP_020918970.1) and the pSTING (GenBank: XP_020932717.1) CTT were modeled using SWISS-MODEL (https://swissmodel.expasy.org). The molecular docking between pIKKε and pSTING CTT was performed *via* the GRAMM-X Protein–Protein Docking Web Server, version 1.2.0 (http://vakser.compbio.ku.edu/resources/gramm/grammx). Finally, the interfaces of the protein complex were analyzed and shown using PyMOL (https://pymol.org/2/).

### Confocal fluorescence microscopy

The 3D4/21 cells in 24-well plate were transfected with indicated plasmids and then fixed with 4% paraformaldehyde for 30 min. The fixed cells were permeabilized with 0.5% Triton X-100 for 20 min. Next, the cell nuclei were stained with 4′,6-diamidino-2-phenylindole for 15 min. The images were visualized by laser-scanning confocal microscope (Leica SP8).

### Statistical analysis

All the results were the representatives of two or three similar experiments. The data in bar graphs were presented as the mean ± SD and analyzed using GraphPad Prism 8.0 (GraphPad Software, Inc) where *p* <0.05 was considered statistically significant as determined by the Student's *t* test. Whereas the *p* >0.05 was considered as not statistically significant.

## Data availability

The Supporting information including Western blotting raw data are available online.

## Supporting information

This article contains supporting information.

## Conflict of interest

The authors declare that they have no conflicts of interest with the contents of this article.

## References

[1] Kumar H., Kawai T., Akira S. (2011). Pathogen recognition by the innate immune system. Int. Rev. Immunol..

[2] Cui J., Chen Y., Wang H.Y., Wang R.F. (2014). Mechanisms and pathways of innate immune activation and regulation in health and cancer. Hum. Vaccin. Immunother..

[3] Takeuchi O., Akira S. (2010). Pattern recognition receptors and inflammation. Cell.

[4] Kell A.M., Gale M. (2015). RIG-I in RNA virus recognition. Virology.

[5] Dempsey A., Bowie A.G. (2015). Innate immune recognition of DNA: a recent history. Virology.

[6] Ablasser A., Goldeck M., Cavlar T., Deimling T., Witte G., Röhl I. (2013). cGAS produces a 2'-5'-linked cyclic dinucleotide second messenger that activates STING. Nature.

[7] Ishikawa H., Barber G.N. (2008). STING is an endoplasmic reticulum adaptor that facilitates innate immune signalling. Nature.

[8] Hopfner K.P., Hornung V. (2020). Molecular mechanisms and cellular functions of cGAS-STING signalling. Nat. Rev. Mol. Cell Biol..

[9] Zhao B., Du F., Xu P., Shu C., Sankaran B., Bell S.L. (2019). A conserved PLPLRT/SD motif of STING mediates the recruitment and activation of TBK1. Nature.

[10] Zhang C., Shang G., Gui X., Zhang X., Bai X.C., Chen Z.J. (2019). Structural basis of STING binding with and phosphorylation by TBK1. Nature.

[11] Tanaka Y., Chen Z.J. (2012). STING specifies IRF3 phosphorylation by TBK1 in the cytosolic DNA signaling pathway. Sci. Signal..

[12] Cai X., Chiu Y.H., Chen Z.J. (2014). The cGAS-cGAMP-STING pathway of cytosolic DNA sensing and signaling. Mol. Cell.

[13] Schoggins J.W., MacDuff D.A., Imanaka N., Gainey M.D., Shrestha B., Eitson J.L. (2014). Pan-viral specificity of IFN-induced genes reveals new roles for cGAS in innate immunity. Nature.

[14] Abe T., Barber G.N. (2014). Cytosolic-DNA-mediated, STING-dependent proinflammatory gene induction necessitates canonical NF-κB activation through TBK1. J. Virol..

[15] Fitzgerald K.A., McWhirter S.M., Faia K.L., Rowe D.C., Latz E., Golenbock D.T. (2003). IKKepsilon and TBK1 are essential components of the IRF3 signaling pathway. Nat. Immunol..

[16] Sharma S., tenOever B.R., Grandvaux N., Zhou G.P., Lin R., Hiscott J. (2003). Triggering the interferon antiviral response through an IKK-related pathway. Science.

[17] Hacker H., Karin M. (2006). Regulation and function of IKK and IKK-related kinases. Sci. STKE.

[18] Zhang J., Tian M., Xia Z., Feng P. (2016). Roles of IkappaB kinase epsilon in the innate immune defense and beyond. Virol. Sin..

[19] Liu H., Ye G., Liu X., Xue M., Zhou Q., Zhang L. (2022). Vimentin inhibits type I interferon production by disrupting the TBK1-IKKε-IRF3 axis. Cell Rep..

[20] Balka K.R., Louis C., Saunders T.L., Smith A.M., Calleja D.J., D'Silva D.B. (2020). TBK1 and IKKε act redundantly to mediate STING-induced NF-κB responses in myeloid cells. Cell Rep..

[21] Wegner J., Hunkler C., Ciupka K., Hartmann G., Schlee M. (2023). Increased IKKϵ protein stability ensures efficient type I interferon responses in conditions of TBK1 deficiency. Front. Immunol..

[22] Luo J., Zhang J., Ni J., Jiang S., Xia N., Guo Y. (2022). The African swine fever virus protease pS273R inhibits DNA sensing cGAS-STING pathway by targeting IKKε. Virulence.

[23] Jiang S., Luo J., Zhang Y., Cao Q., Wang Y., Xia N. (2022). The porcine and Chicken innate DNA sensing cGAS-STING-IRF signaling axes exhibit differential species specificity. J. Immunol..

[24] Prins K.C., Cárdenas W.B., Basler C.F. (2009). Ebola virus protein VP35 impairs the function of interferon regulatory factor-activating kinases IKKepsilon and TBK-1. J. Virol..

[25] Fang R., Jiang Q., Zhou X., Wang C., Guan Y., Tao J. (2017). MAVS activates TBK1 and IKKε through TRAFs in NEMO dependent and independent manner. PLoS Pathog..

[26] Fang P., Fang L., Xia S., Ren J., Zhang J., Bai D. (2020). Porcine deltacoronavirus accessory protein NS7a antagonizes IFN-β production by competing with TRAF3 and IRF3 for binding to IKKε. Front. Cell. Infect. Microbiol..

[27] Yum S., Li M., Fang Y., Chen Z.J. (2021). TBK1 recruitment to STING activates both IRF3 and NF-κB that mediate immune defense against tumors and viral infections. Proc. Natl. Acad. Sci. U. S. A..

[28] Kawai T., Akira S. (2008). Toll-like receptor and RIG-I-like receptor signaling. Ann. N. Y. Acad. Sci..

[29] Rehwinkel J., Gack M.U. (2020). RIG-I-like receptors: their regulation and roles in RNA sensing. Nat. Rev. Immunol..

[30] Zheng W., Xia N., Zhang J., Cao Q., Jiang S., Luo J. (2022). African swine fever virus structural protein p17 inhibits cGAS-STING signaling pathway through interacting with STING. Front. Immunol..

[31] Lv L., Bai J., Gao Y., Jin L., Wang X., Cao M. (2021). Peroxiredoxin 1 interacts with TBK1/IKKepsilon and negatively regulates pseudorabies virus propagation by promoting innate immunity. J. Virol..

[32] Liu S., Cai X., Wu J., Cong Q., Chen X., Li T. (2015). Phosphorylation of innate immune adaptor proteins MAVS, STING, and TRIF induces IRF3 activation. Science.

[33] Zhang X., Bai X.C., Chen Z.J. (2020). Structures and mechanisms in the cGAS-STING innate immunity pathway. Immunity.

[34] Zhang K., Zhang Y., Xue J., Meng Q., Liu H., Bi C. (2019). DDX19 inhibits type I interferon production by disrupting TBK1-IKKepsilon-IRF3 interactions and promoting TBK1 and IKKepsilon degradation. Cell Rep..

[35] Zhang N., Shi H., Yan M., Liu G. (2021). IFIT5 negatively regulates the type I IFN pathway by disrupting TBK1-IKKε-IRF3 signalosome and degrading IRF3 and IKKε. J. Immunol..

[36] Jiang S., Xia N., Luo J., Zhang Y., Cao Q., Zhang J. (2022). The porcine cyclic GMP-AMP synthase-STING pathway exerts an unusual antiviral function independent of interferon and autophagy. J. Virol..

[37] Xu Y., Ye M., Zhang Y., Sun S., Luo J., Jiang S. (2021). Screening of porcine innate immune adaptor signaling revealed several anti-PRRSV signaling pathways. Vaccines (Basel).

