# Supplementary material for: Porcine IKKε is involved in the STING-induced type I IFN antiviral response of the cytosolic DNA signaling pathway
Source: J Biol Chem. 2023 Sep 1;299(10):105213. doi: 10.1016/j.jbc.2023.105213 (PMC10520887; doi:10.1016/j.jbc.2023.105213)
Supplement: Supporting Tables S1–S3 [file mmc1.docx]

**Supplementary Table 1: The PCR primers used for gene cloning and mutation**

| **Primer names** | **Primer sequences** |
| --- | --- |
| Cloning PCR primers | |
| **EGFP-N1-pIKKε** | F: AGCTGTACAAGTCCGGACTC*AGATCT*ATGCAGAGCACCGTCA |
|  | R: CCGGTGGATCCCGGGCCCGC*GGTACC*TCAGATGTCAGGAGCC |
| **pCAGGS-pIKKε** | F: GGCAAA*GAATTC*ATGCAGAGCACCGTC |
|  | R: CTGCTC*GATATC*GATGTCAGGAGCCGC |
| **mCherry-C1- pIKKε** | F: AGCTGTACAAGTCCGGACTC*AGATCT*ATGCAGAGCACCGTCA |
|  | R: CCGGTGGATCCCGGGCCCGC*GGTACC*TCAGATGTCAGGAGCC |
| **pIKKε-KD** | F:GCTACCGGACTC*AGATCT*ATGCAGAGCACCGTCAATTACCTG |
|  | R:ATCCCGGGCCCGC*GGTACC*GTGTCACTGGTCTCCGCAAAGAA |
| **pIKKε-SDD** | F: AGCGCTACCGGACTC*AGATCT*ATGAGCGTGGCCAGCGAGATC |
|  | R: TGGATCCCGGGCCCGC*GGTACC*GTAAGGATCTGGCTGAGGCT |
| **pENTR4-IKKε** | F: CCAATTCA*GTCGAC*ATGCAGAGCA |
|  | R: GCTGGT*GATATC*GATGTCAGAGC |
| **mCherry-C1-pTBK1** | F: TCCGGACTC*AGATCT*ATGCAGAGCACTTCT |
|  | R: GCCCGC*GGTACC*CTAAAGACAGTCAACATTGCG |
| **pCAGGS-pTBK1** | F: CATCATTTTGGCAAA*GAATTC*ATGCAGAGCACTTCTAATCAT |
|  | R: GTATGGGTAGCTGGT*GATATC*AAGACAGTCAACATTGCGAAG |
| **pGEX-6p-1-pSTING** | F: GGATCCCCG*GAATTC*ATGCCCTACTCCAGC |
|  | R: GCCGCTCGA*GTCGAC*GAAGATATCTGAGCG |
| **pIRF3 1-114** | F: AAGTCCGGACTC*AGATCT*ATGGGAACTCAGAAGCCTCGG |
|  | R: GGATCCCGGGCCCGC*GGTACC*CTAAACTCCTGAGGTCAC |
| **pIRF3 187-378** | F: AAGTCCGGACTC*AGATCT*GAAAACCCACTGAAGCAG |
|  | R: GGATCCCGGGCCCGC*GGTACC*CTACCGCGCCATGTC |
| **pIRF3 378-421** | F: CTGTACAAGTCCGGACTC*AGATCT*GACGGGGGCGCCTCC |
|  | R: GGATCCCGGGCCCGC*GGTACC*CTAGAAATCCATGTCCTC |
| **pIRF3 1-378** | F: AAGTCCGGACTC*AGATCT*ATGGGAACTCAGAAGCCTCGG |
|  | R: GGATCCCGGGCCCGC*GGTACC*CTACCGCGCCATGTC |
| **pIRF3 187-421** | F: AAGTCCGGACTC*AGATCT*GAAAACCCACTGAAGCAG |
|  | R: GGATCCCGGGCCCGC*GGTACC*CTAGAAATCCATGTCCTC |
|  | |
| Mutation PCR primers | |
| **pSTING 191-338** | F:GACTGCAGAATTCTCACTCCCTTTCCTCCTGC |
|  | R:GCAGGAGGAAAGGGAGTGAGAATTCTGCAGTC |
| **pSTING L373A** | F: TATCTGAGCGGGCTGGAAGAGGCTGTTCCATGCC |
|  | R: GGCATGGAACAGCCTCTTCCAGCCCGCTCAGATA |
| **Δ TBM** | F: GCAGAATTCTCAGAAGATCATGCCACTGATGAGGAG |
|  | R: CTCCTCATCAGTGGCATGATCTTCTGAGAATTCTGC |
|  |  |

Note: The p denotes porcine. The restriction enzyme sites were *italic* and underlined. The pCAGGS-pIKKε and mCherry-C1-pIKKε were made with T4 DNA Ligase whereas other clones were ligated with 2×MultiF Seamless Assembly Mix. The pSTING 191-338 was made by mutation PCR from the template pSTING 191-378. Both pSTING ΔTBM and pSTING CTT ΔTBM were made by mutation PCR with the same primers from templates pSTING and pSTING CTT, respectively.

**Supplementary Table 2: Primers for RT-qPCR in this study**

| **Primer names** | **Primer sequences** |
| --- | --- |
| **pIFN-β** | F: TGAGCATTCTGCAGTACCTGA |
|  | R: CCGGAGGTAATCTGTAAGTCTGT |
| **pISG56** | F: ATGGGAGTTGGTCATTCAAGA |
|  | R:CAGGTGTTTCACATAGGCCA |
| **pIL-8** | F: CTGCAGTTCTGGCAAGAGTAAGT |
|  | R: CACTCTCAATCACTCTCAGTTCCT |
| **pβ-actin** | F: ATGAAGATCAAGATCATCGCG |
|  | R:TCGTACTCCTGCTTGCTGATC |
| **HSV1 gB** | F: TTCTGCAGCTCGCACCAC |
|  | R: GGAGCGCATCAAGACCACC |

Note: The p denotes porcine. The pβ-actin serves as the house keeping gene.

**Supplementary Table 3: The CRISPR gRNA encoding DNA sequences and PCR primer for porcine IKKε gene**

| **gRNA names** | **gRNA encoding DNA sequences** |
| --- | --- |
| **pIKKε gRNA1-F**  **pIKKε gRNA1-R**  **pIKKε gRNA-PCR-F**  **pIKKε gRNA-PCR-R** | CACCGATTTCGGGCCTTGTACACGC  AAACGCGTGTACAAGGCCCGAAATC  **PCR primer sequences**  GGAGCAGGAGATGCAGAGCA  ACAGAAGTGTCATTCCGAGGGT |

Note: The p denotes porcine.
